# Pancreatic cancer cells resistance to gemcitabine: the role of MUC4 mucin

**DOI:** 10.1038/sj.bjc.6605285

**Published:** 2009-09-08

**Authors:** S Bafna, S Kaur, N Momi, S K Batra

**Affiliations:** 1 Department of Biochemistry and Molecular Biology, University of Nebraska Medical Center, Omaha, NE 68198-5870, USA

**Keywords:** MUC4, pancreatic cancer, chemotherapy, resistance

## Abstract

**Background:**

A major obstacle to the successful management of pancreatic cancer is to acquire resistance to the existing chemotherapeutic agents. Resistance to gemcitabine, the standard first-line chemotherapeutic agent for advanced and metastatic pancreatic cancer, is mainly attributed to an altered apoptotic threshold in the pancreatic cancer. The MUC4 transmembrane glycoprotein is aberrantly overexpressed in the pancreatic cancer and recently, has been shown to increase pancreatic tumour cell growth by the inhibition of apoptosis.

**Methods:**

Effect of MUC4 on pancreatic cancer cells resistance to gemcitabine was studied in MUC4-expressing and MUC4-knocked down pancreatic cancer cell lines after treatment with gemcitabine by Annexin-V staining, DNA fragmentation assay, assessment of mitochondrial cytochrome *c* release, immunoblotting and co-immunoprecipitation techniques.

**Results:**

Annexin-V staining and DNA fragmentation experiment demonstrated that MUC4 protects CD18/HPAF pancreatic cancer cells from gemcitabine-induced apoptosis. In concert with these results, MUC4 also attenuated mitochondrial cytochrome *c* release and the activation of caspase-9. Further, our results showed that MUC4 exerts anti-apoptotic function through HER2/extracellular signal-regulated kinase-dependent phosphorylation and inactivation of the pro-apoptotic protein Bad.

**Conclusion:**

Our results elucidate the function of MUC4 in imparting resistance to pancreatic cancer cells against gemcitabine through the activation of anti-apoptotic pathways and, thereby, promoting cell survival.

Pancreatic adenocarcinoma is among the most common causes for cancer-related deaths in western countries (Keighley, 2003). It is one of the neoplasms with an extremely poor prognosis because of its aggressive invasion, early metastasis, resistance to existing chemotherapeutic agents and radiation therapy (Bardeesy and DePinho, 2002). Despite an enormous amount of effort spent in the development of chemotherapies for pancreatic cancer, these are effective only in a small proportion of patients. Gemcitabine has become the standard first-line chemotherapeutic agent for the advanced and metastatic pancreatic cancer, with marginal survival advantage, and amelioration of disease-related symptoms (El-Rayes and Philip, 2003; Pino *et al*, 2004). In contrast, resistance to gemcitabine has been increasing in recent years, and the effectiveness of gemcitabine has been reduced to <20% (Wheatley and McNeish, 2005). It is considered that resistance to gemcitabine treatment is mainly attributed to an altered apoptotic threshold in pancreatic cancer cells (Schniewind *et al*, 2004).

MUC4, a membrane-bound mucin, is involved in the regulation of cell proliferation and inhibition of apoptosis (Chaturvedi *et al*, 2007). To date, the aberrant overexpression of MUC4 has been reported in pancreatic malignancies, but not in the normal pancreas, which has made MUC4 a promising therapeutic target for anti-cancer adjuvant therapies (Andrianifahanana *et al*, 2001). Recently, we have shown that the overexpression of MUC4 in mouse embryonic fibroblast cells confers oncogenic transformation (Bafna *et al*, 2008). In addition, studies by overexpression and down-regulation of MUC4 in various pancreatic cancer cells showed its involvement in the development and progression of pancreatic cancer (Singh *et al*, 2004; Chaturvedi *et al*, 2007; Moniaux *et al*, 2007). Importantly, our recent studies have revealed that MUC4 interacts with HER2, a member of epidermal growth factor (EGF) receptor family and regulates its expression by post-translational mechanisms (Chaturvedi *et al*, 2008). HER2 is an established oncoprotein and is involved in growth and malignant properties of the cancer cells through activation of various intracellular signalling pathways (Hsieh and Moasser, 2007). It has been shown that the EGF protects prostate cancer cells from apoptosis by phosphorylating apoptotic protein Bad through extracellular signal-regulated kinase (ERK) activation (Sastry *et al*, 2006). In our earlier studies, MUC4 has been shown to increase the phosphorylation of ERK by stabilization of the expression of HER2 in pancreatic cancer cells (Bafna *et al*, 2008; Chaturvedi *et al*, 2008). These findings indicate that MUC4 might be responsible for resistance to gemcitabine treatment by alteration of apoptotic threshold in pancreatic cancer cells.

In this study, we performed a set of experiments to define the function of MUC4 in the activation of anti-apoptotic pathways in response to gemcitabine treatment of pancreatic cancer cells. Earlier MUC4-down-regulated CD18/HPAF cells (CD18/HPAF/siMUC4) and scrambled siRNA-transfected CD18/HPAF cells (CD18/HPAF/Scr) were treated with gemcitabine for 24 and 48 h and extent of apoptosis was measured. Annexin-V staining showed that MUC4 inhibited gemcitabine-induced apoptosis of CD18/HPAF/Scr pancreatic cancer cells. CD18/HPAF/Scr cells also showed reduced DNA fragmentation, a hallmark of apoptosis, compared with CD18/HPAF/siMUC4 cells. In concert with this, the release of mitochondrial cytochrome *c* and the activation of caspase-9 were attenuated in MUC4-expressing CD18/HPAF/Scr cells compared with MUC4-down-regulated CD18/HPAF/siMUC4 cells. Interestingly, the expression of MUC4 was associated with the increased level of phospho-HER2 and -ERK, which further leads to deactivation of apoptotic protein Bad through enhancing its phosphorylation. Taken together, these findings indicate that aberrant overexpression of MUC4 in pancreatic cancer contributes resistance to chemotherapeutic agent gemcitabine by activation of MUC4-HER2-mediated anti-apoptotic pathway.

## Materials and methods

### Cell culture

Earlier generated MUC4-knocked down CD18/HPAF pancreatic cancer cell line (CD18/HPAF/siMUC4) and control CD18/HPAF (CD18/HPAF/Scr) cells (Chaturvedi *et al*, 2007) were cultured in DMEM supplemented with 10% foetal bovine serum and antibiotics (penicillin and streptomycin 100 *μ*g ml^–1^). Cells were grown at 37 °C with 5% CO_2_ in a humidified atmosphere.

### Measurement of apoptosis

Apoptosis was measured by using the Annexin-V Fluos staining kit (Roche Diagnostics, Indianapolis, IN, USA). For this, 1.5 × 10^6^ cells each of CD18/siMUC4 and control CD18/Scr were cultured in 10 cm petridishes followed by overnight incubation at 37 °C. The cells were then treated with 1 *μ*M gemcitabine (Symon *et al*, 2002) in 10% DMEM for 24 and 48 h, respectively, followed by 24 h incubation in 10% DMEM. The induction of apoptosis and necrosis was measured by staining the cells with Annexin-V and propidium iodide solution, followed by fluorescence-activated cell sorting analysis (FACs).

### Assessment of mitochondrial cytochrome *c* release

Cytosolic fraction was prepared as described by Kharbanda *et al* (1997). Briefly, cells were washed twice with PBS, and the pellet of 1.5 × 10^6^ was suspended in 1 ml of ice-cold buffer A (20 mM Hepes, pH 7.5/1.5 mM, MgCl_2_/10 mM, KCl/1 mM, EDTA/1 mM, EGTA/1 mM, DTT/0.1 mM, phenylmethylsulfonyl fluoride and 1 × protease inhibitor cocktail (Roche)) containing 250 mM sucrose. The cells were homogenized by douncing three times in a dounce homogenizer with a sandpaper-polished pestle. After centrifugation for 5 min at 4°C, the supernatants were ultracentrifuged at 105 000 × *g* for 30 min at 4°C. The resulting supernatant was used as the soluble cytosolic fraction. Protein concentrations in the soluble cytosolic fractions were determined using a Bio-Rad_D/C_ protein estimation kit. The same amount of protein from the cytosolic fractions of CD18/Scr and CD18/siMUC4 cells were used to quantify the release of cytochrome *c* from mitochondria, using a commercially available cytochrome *c* ELISA kit (Calbiochem, San Diego, CA, USA) according to the manufacturer's instructions.

### DNA fragmentation assay

CD18/HPAF/Scr and CD18/HPAF/siMUC4 cells were cultured in 10% DMEM with and without 1 *μ*M of gemcitabine. Treated cells were washed twice with PBS, and DNA was extracted using Gentra's Puregene DNA Isolation Kit (Qiagen, Valencia, CA, USA) protocol. A measure of 5 *μ*g of isolated DNA was resolved on a 1% agarose gel.

### Immunoblot analysis

Gemcitabine-treated and -untreated CD18/HPAF/Scr and CD18/HPAF/siMUC4 cells were processed for protein extraction and western blotting using standard procedures. Cell lysates were prepared as described earlier (Bafna *et al*, 2008). Protein concentrations were determined using a Bio-Rad_D/C_ protein estimation kit. For MUC4, the proteins (20 *μ*g) were resolved by electrophoresis on a 2% SDS–agarose gel under reducing conditions. For *β*-actin, HER2, p^1248^HER2, ERK1/2 and pERK1/2, SDS–PAGE (10%) was performed under similar conditions. For Bad, pBad and caspase-9, SDS–PAGE (15%) was performed under similar conditions. Resolved proteins were transferred onto the polyvinylidene difluoride membrane and blocked in 5% non-fat milk in phosphate-buffered saline (PBS) for 2 h and subjected to the standard immunodetection procedure using specific antibodies. For *β*-actin immunodetection, anti-*β*-actin mouse monoclonal antibody (Sigma, St Louis, MO, USA) in dilution of 1:5000 (used as internal control) was used; and for MUC4 immunodetection, anti-MUC4 mouse monoclonal antibody (8G7, generated in our laboratory) in dilution of 1:1000 was used. For HER2, p^1248^HER2, ERK, pERK and caspase-9 immunodetection, anti-ErbB2 rabbit polyclonal antibody (Santa Crutz Biotecnology, Santa Cruz, CA, USA), anti-p^1248^ErbB2 rabbit polyclonal antibody (Upstate, San Francisco, CA, USA), anti-ERK1/2 rabbit polyclonal antibody (Santa Crutz Biotecnology), anti-pERK1/2 rabbit polyclonal antibody (Cell Signaling Technology Inc, Danvers, MA, USA) and anti-caspase-9 mouse monoclonal antibody (Cell Signaling Technology Inc) in dilution of 1:1000 were used, respectively. For 14-3-3, Bad and pBad immunodetection, anti-14-3-3 mouse monoclonal antibody in dilution of 1:250 (Santa Crutz Biotecnology), anti-Bad mouse monoclonal antibody in dilution of 1:500 (Santa Crutz Biotecnology) and anti-pBad goat polyclonal antibody in dilution of 1:200 (Santa Crutz Biotecnology) were used, respectively. The membranes were incubated for 4 h at room temperature, followed by 6 × 10 min washes in TBST (50 mM Tris–HCl, pH 7.4, 150 mM NaCl and 0.05% Tween-20). Further, the membranes were incubated in Horseradish peroxidase-conjugated secondary antibodies (Amersham Biosciences, Buckinghamshire, UK) (diluted at 1:2000 in PBS) for 1 h at room temperature, followed by four washes in TBST. The blots were processed with ECL Chemiluminescence kit (Amersham Biosciences), and the signal was detected by exposing the processed blots to X-ray films (Biomax Films, Kodak, NY, USA).

### Co-immunoprecipitation

Cells were grown to 50–60% confluency and treated with 1 *μ*M gemcitabine for 48 h in 5% CO_2_ incubator at 37°C. Cells were washed once with ice-cold PBS and then lysed in extraction buffer, which contains 1% Triton X-100 in lysis buffer (150 mM NaCl, 2 mM EDTA, 50 mM Tris–Cl (pH 8.0), 1 mM NaF, 1 mM sodium orthovanadate, 1 mM PMSF, 5 *μ*g of aprotinin per ml and 5 *μ*g of leupeptin per ml) for 25–35 min at 4°C. The lysates were centrifuged at 16 000 × *g* for 30 min at 4°C. Protein concentrations were determined using a Bio-Rad_D/C_ protein estimation kit. Equal amounts of protein cell lysates were incubated overnight with anti-14-3-3 mAbs or IgG in a 500-*μ*l total volume. Protein G-Sepharose beads (Oncogene Research, Boston, MA, USA) were added to the lysate–antibody mix and incubated on a rotating platform for 2.5–3.5 h at 4°C, followed by three to four washes with the lysis buffer. The immunoprecipitates or total cell lysates were then immunoblotted with anti-14-3-3 mouse monoclonal antibody and anti-pBad goat polyclonal antibody.

## Results

### MUC4 confers resistance to gemcitabine-induced apoptosis in pancreatic cancer cells

**Figure 1:**
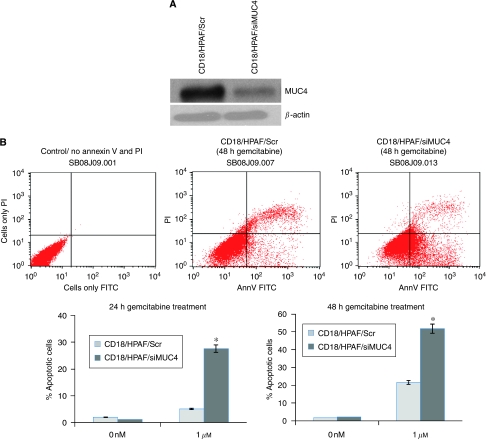
Assessment of function of MUC4 in resistance to gemcitabine-induced apoptosis in CD18/HPAF pancreatic cancer cells. MUC4 was down-regulated in CD18/HPAF cells (CD18/HPAF/siMUC4) by using siRNA technology earlier in our laboratory (Chaturvedi *et al*, 2007). Scrambled siRNA-transfected CD18/HPAF cells (CD18/HPAF/Scr) were used as a control. (**A**) Western blot analysis was carried out to measure the expression of MUC4. A total of 20 *μ*g protein from cell extracts was resolved by electrophoresis on a 2% SDS–agarose gel, transferred to polyvinylidene difluoride membrane and immunoblotted with anti-MUC4 mAb (8G7). The membrane was then probed with horseradish peroxidase-labelled goat anti-mouse immunoglobulin. Immunoblot of *β*-actin, obtained from 10% SDS–PAGE, was used as an internal control. Further, these cells were used for measurement of apoptosis. (**B**) Cells were seeded in 10 cm petridishes and treated with 1 *μ*M gemcitabine for 24 and 48 h. The cells were then stained with PI and Annexin-V and percentage of cells undergoing apoptosis was analysed by FACs. Bottom left quadrant, being negative for both Annexin-V and propidium iodide, shows the live cells; bottom right quadrant, being Annexin-V positive and propidium iodide negative, shows the early apoptotic cells; top right quadrant, being both propidium iodide positive and Annexin-V positive, shows the late apoptotic or necrotic cells. The results are expressed as the percentage (Mean±s.e.) of apoptotic cells for CD18/HPAF/siMUC4 cells compared with CD18/HPAF/Scr cells after treating the cells with 1 *μ*M gemcitabine for 24 and 48 h. MUC4 protects CD18/HPAF cells from gemcitabine-induced apoptosis.

Membrane-bound mucin MUC1 and rat Muc4 have been shown to inhibit apoptosis induced by multiple insults in rat 3Y1 fibroblast cells and human melanoma and breast cancer cells, respectively (Raina *et al*, 2004; Workman *et al*, 2009). Anti-apoptotic function of MUC4 in pancreatic cancer cells in response to serum starvation has also been observed earlier in our laboratory (Chaturvedi *et al*, 2007). Further, an altered apoptotic threshold is considered to be one of the major attribute for the development of resistance to gemcitabine treatment in pancreatic cancer cells (Schniewind *et al*, 2004). Therefore, to determine the function of MUC4 in the development of resistance to gemcitabine in pancreatic cancer cells, we assessed the effect of MUC4 down-regulation on gemcitabine-induced apoptosis in CD18/HPAF pancreatic cancer cells. MUC4 was stably down-regulated in CD18/HPAF pancreatic cancer cells, which express high level of MUC4, by MUC4 siRNA and resulting stable transfectant pool (CD18/HPAF/siMUC4) was quantitated for MUC4 expression by immunoblot analysis (Figure 1A). Scrambled siRNA-transfected CD18/HPAF cells (CD18/HPAF/Scr) were used as a control. These cell lines were further used to study the effect of MUC4 on gemcitabine-induced apoptosis. To analyse the apoptotic index, the MUC4-overexpressing and MUC4-silenced cells were treated with gemcitabine for 24 and 48 h and the extent of apoptosis was determined by Annexin-V and propidium iodide staining followed by flow cytometric analysis. The results showed that gemcitabine treatment at both time points was directly associated with apoptosis in CD18/HPAF/siMUC4 cells and this response was suppressed in CD18/HPAF/Scr cells (Figure 1B).

**Figure 2:**
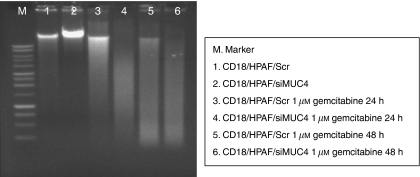
Determination of DNA fragmentation in response to gemcitabine treatment of CD18/HPAF/Scr and CD18/HPAF/siMUC4 cells. Cells were seeded in 10 cm petridishes and treated without and with 1 *μ*M gemcitabine for 24 and 48 h. Further, genomic DNA was isolated and 5 *μ*g of isolated DNA resolved on 1% agarose gel. MUC4-expressing CD18/HPAF/Scr cells exhibit reduced DNA fragmentation compared with CD18/HPAF/siMUC4 cells.

Further, DNA fragmentation, which is a hallmark of apoptosis, was checked in these cell lines. For this, genomic DNA was isolated from CD18/HPAF/Scr and CD18/HPAF/siMUC4 cells before and after treatment with gemcitabine and resolved on 1% agarose gel. Our results showed that MUC4-expressing CD18/HPAF/Scr exhibited reduced DNA fragmentation compared with MUC4-silenced CD18/HPAF/siMUC4 cells (Figure 2). These observations corroborated the finding that MUC4 protects CD18/HPAF pancreatic cancer cells from gemcitabine-induced apoptosis.

### MUC4 blocks activation of intrinsic apoptotic pathway

**Figure 3:**
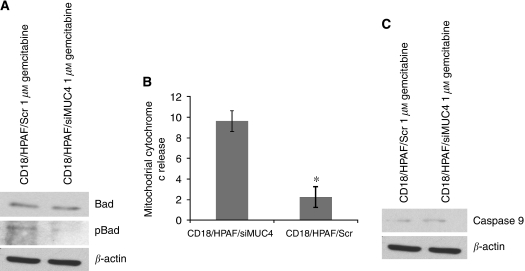
Assessment of intrinsic apoptotic pathway in CD18/HPAF/Scr and CD18/HPAF/siMUC4 cells in response to gemcitabine treatment. (**A**) Cells were seeded in 10 cm petridishes and treated with 1 *μ*M gemcitabine for 48 h as described in methodology. A total of 50 *μ*g protein from each cell extract was resolved by SDS–PAGE (15%), followed by immunobloting with anti-pBad, anti-Bad and anti-*β*-actin (internal control) antibodies. pBad protein level was more in CD18/HPAF/Scr cells compared with CD18/HPAF/siMUC4 cells. (**B**) Cytosolic fractions were prepared from 1 *μ*M gemcitabine-treated CD18/HPAF/Scr and CD18/HPAF/siMUC4 cells. The amount of cytochrome *c* protein from each fraction was then measured with a commercially available cytochrome *c* ELISA kit. Level of cytochrome *c* in the cytosol of CD18/HPAF/siMUC4 was more compared with CD18/HPAF/Scr cells. (**C**) A total of 20 *μ*g protein from each cell lines treated with 1 *μ*M gemcitabine for 48 h was resolved by SDS–PAGE (10%), followed by immunobloting with antibodies against cleaved caspase-9 and *β*-actin (internal control). CD18/HPAF/siMUC4 cells showed more cleaved caspase-9 compared with CD18/HPAF/Scr cells. These findings indicate up-regulation of intrinsic apoptotic pathway in CD18/HPAF/Scr cells.

The balance among the pro and anti-apoptotic members of the Bcl-2 family proteins has a central function in the regulation of intrinsic apoptotic pathway by controlling the activation of mitochondrial cytochrome *c* release in the cytosol. The mitochondrial associated anti-apoptotic proteins Bcl-2 and Bcl-X_L_ suppress intrinsic mitochondrial apoptotic pathway, whereas pro-apoptotic proteins, such as Bad, translocate to mitochondria in response to apoptotic signals and interact with and deactivate Bcl-2 and Bcl-X_L_ (Yang *et al*, 1995; Thomadaki and Scorilas, 2006). The pro-apoptotic activity of Bad is suppressed by its phosphorylation on serine residues in response to survival signalling cascades. As increase in phospho-Bad and Bcl-X_L_ protects against apoptosis; to assess the effect of MUC4, we examined expression of these proteins in our cell models CD18/HPAF/Scr and CD18/HPAF/siMUC4 after treatment with gemcitabine. We found that MUC4 markedly increased level of phosphorylated Bad in CD18/HPAF/Scr cells (Figure 3A). No difference was observed in Bad protein levels between CD18/HPAF/Scr and CD18/HPAF/siMUC4 cells (Figure 3A). MUC1 and rat Muc4 has been shown to decrease apoptosis in response to various insults also by enhancing the expression of Bcl-X_L_ (Raina *et al*, 2006; Thomadaki and Scorilas, 2006; Workman *et al*, 2009). In contrast, we did not observe any difference in Bcl-X_L_ protein levels after gemcitabine treatment of CD18/HPAF/Scr and CD18/HPAF/siMUC4 cells (data not shown).

Further, to determine the downstream effect of phosphorylated Bad on activation of the intrinsic mitochondrial apoptotic pathway, we examined the release of mitochondrial cytochrome *c* into cytosol and the activation of caspase-9. In response to gemcitabine treatment, mitochondrial cytochrome *c* release was significantly increased in MUC4-silenced CD18/HPAF/siMUC4 cells compared with CD18/HPAF/Scr cells (Figure 3B). In concert with this, the level of cleaved caspase-9 protein was also enhanced in CD18/HPAF/siMUC4 cells (Figure 3C). These observations suggest that MUC4 blocks activation intrinsic mitochondrial apoptotic pathway in CD18/HPAF pancreatic cancer cells in response to gemcitabine treatment.

### MUC4 facilitates sequestration of Bad in the cytosol

**Figure 4:**
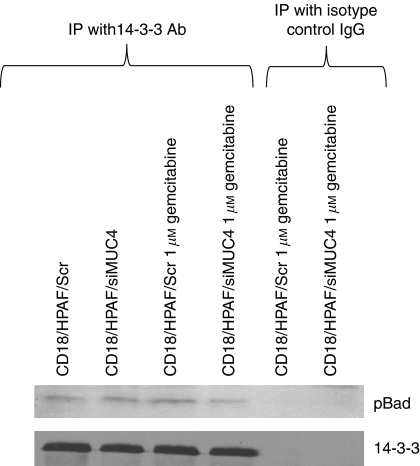
Analysis of interaction between pBad and 14-3-3 proteins in CD18/HPAF/Scr and CD18/HPAF/siMUC4 cells in response to gemcitabine treatment by co-immunoprecipiation assay. Lysates from 1 *μ*M gemcitabine-treated and -untreated CD18/HPAF/Scr and CD18/HPAF/siMUC4 cells were used for immunoprecipitation with mouse anti-14-3-3 antibody. The immunoprecipitates were electrophoretically resolved on a 15% polyacrylamide gel and immunoblotted with anti-14-3-3 and anti-pBad antibodies. The mouse IgG was used as isotype control for co-immunoprecipitation study. CD18/HPAF/Scr cells showed enhanced precipitation of pBad with 14-3-3 proteins in response to gemcitabine treatment compared with CD18/HPAF/siMUC4 cells.

Phosphorylation of Bad promotes its interaction with the scaffolding protein 14-3-3 and prevents its interaction with anti-apoptotic Bcl-X_L_ protein, leading to its sequestration in the cytosol and inhibition of its pro-apoptotic activity (Thomadaki and Scorilas, 2006). We found that MUC4 increases phosphorylation of Bad in CD18/HPAF/Scr cells in response to gemcitabine treatment. Here, we determined that whether increased phosphorylation of Bad was associated with the increased binding with 14-3-3 proteins. For this, we performed co-immunoprecipitation experiment for pBad and 14-3-3 proteins. Our data showed that pBad was pulled down in 14-3-3 immunoprecipitates in 1 *μ*M gemcitabine-treated CD18/HPAF/Scr cells (Figure 4). Pull down of pBad in 14-3-3 immunoprecipitates was decreased in CD18/HPAF/siMUC4 cells after treatment with 1 *μ*M gemcitabine (Figure 4). This suggests that the expression of MUC4 promotes binding of Bad with 14-3-3 proteins and thereby helps in its sequestration in the cytosol.

### MUC4 activates HER2 downstream signalling pathway

**Figure 5:**
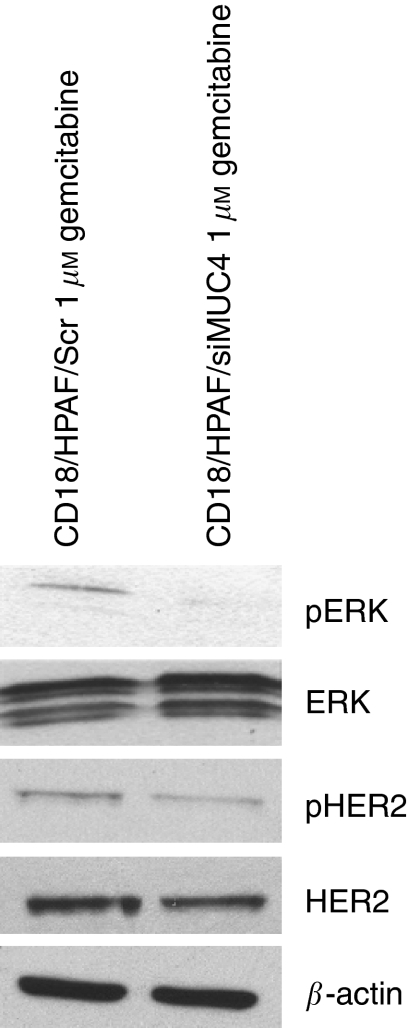
Assessment of activation of HER2 and ERK in CD18/HPAF/Scr and CD18/HPAF/siMUC4 cells in response to gemcitabine treatment. A total of 1.5 × 10^6^ cells were seeded in 10 cm petridishes and treated with 1 *μ*M gemcitabine for 48 h. A measure of 20 *μ*g of whole cell lysate was resolved by SDS–PAGE (10%), followed by immunoblotting with transferred antibodies against HER2, pHER2, ERK, pERK and *β*-actin (internal control). CD18/HPAF/Scr cells showed increased expression of HER2 and also enhanced phosphorylation of HER2 and ERK compared with CD18/HPAF/siMUC4 cells.

Our recent studies have revealed that MUC4 interacts with HER2, a member of EGF receptor family and regulates its expression by post-translational mechanisms (Chaturvedi *et al*, 2008). To determine whether MUC4 exerts its anti-apoptotic function in pancreatic cancer cells through HER2, we examined the expression and activation of HER2 and its downstream signalling proteins. Our results showed increased expression and activation of HER2 in CD18/HPAF/Scr cells compared with CD18/HPAF/siMUC4 cells in response to gemcitabine treatment (Figure 5). Enhanced activation of HER2 was also associated with enhanced activation of ERK (Figure 5). This indicates that MUC4 contributes resistance to chemotherapeutic agent gemcitabine in CD18/HPAF pancreatic cancer cells by activation of the MUC4-HER2-mediated anti-apoptotic pathway.

## Discussion

Our earlier studies have shown the specific and differential expression of MUC4 in pancreatic adenocarcinoma as compared with the normal pancreas or chronic pancreatitis (Andrianifahanana *et al*, 2001). Using MUC4-knockdown and overexpression pancreatic cancer cell models, we have shown that MUC4 potentiates pancreatic tumour cell growth and metastasis by altering the behavioural properties of the tumour cells (Singh *et al*, 2004; Chaturvedi *et al*, 2007; Moniaux *et al*, 2007). Recently, the anti-apoptotic function of MUC4 in pancreatic cancer cells has been observed in our laboratory (Chaturvedi *et al*, 2007). Further, another membrane-bound mucin MUC1 and rat Muc4 have also been shown to inhibit apoptosis induced by multiple insults in rat 3Y1 fibroblast cells and human melanoma and breast cancer cells, respectively (Raina *et al*, 2004; Workman *et al*, 2009). In addition, Muc4 has been shown to impart resistance to the trastuzumab chemotherapeutic agent in breast cancer cells by causing steric interference with the drug (Price-Schiavi *et al*, 2002; Nagy *et al*, 2005). Here, in this study, we explored the function of MUC4 in development of resistance to gemcitabine in pancreatic cancer cells. We have shown that overexpression of MUC4 in pancreatic cancer cells contributes resistance to gemcitabine by activation of an anti-apoptotic pathway. This makes MUC4 an ideal candidate to consider as an important marker for prediction of patient response to therapy.

A membrane-bound mucin MUC1 has been shown to impart resistance to rat fibroblast cells against gemcitabine by induction of the intrinsic apoptotic pathway (Raina *et al*, 2004). Rat Muc4 also provided resistance to chemotherapeutic agent cisplatin in melanoma and breast cancer cells (Workman *et al*, 2009). Consistent with these findings, our data also showed that MUC4 activates intrinsic mitochondrial apoptotic pathways to impart resistance to gemcitabine in CD18/HPAF pancreatic cancer cells. We have observed increased phosphorylation of pro-apoptotic protein Bad in MUC4-expressing CD18/HPAF cells in response to gemcitabine treatment. No change was observed in the expression of anti-apoptotic protein Bcl-X_L_, which has been shown to be a major player in the inhibition of intrinsic apoptotic pathway in response to various insults in earlier studies. The mitochondrial-associated anti-apoptotic proteins Bcl-2 and Bcl-X_L_ suppress intrinsic mitochondrial apoptotic pathway, whereas pro-apoptotic proteins, such as Bad, translocate to mitochondria in response to apoptotic signals, and interact with and deactivate Bcl-2 and Bcl-X_L_ (Yang *et al*, 1995; Thomadaki and Scorilas, 2006). The pro-apoptotic activity of Bad is suppressed by its phosphorylation on serine residues in response to survival signalling cascades. Indeed, phosphorylation at serine residue of Bad is sufficient for binding with scaffolding protein 14-3-3 and thus, inhibits pro-apoptotic function of Bad. MUC4 causes increased phosphorylation of Bad in response to gemcitabine treatment of pancreatic cancer cells, and thereby facilitates increased binding with 14-3-3 proteins. Therefore, Bad will not translocate to mitochondria to deactivate the anti-apoptotic protein Bcl-X_L_. As expected, anti-apoptotic effects of Bad phosphorylation were also associated with decreased mitochondrial cytochrome *c* release in the cytosol for the induction of intrinsic apoptosis. These findings indicate that MUC4-mediated increased phosphorylation of Bad is sufficient to protect pancreatic cancer cells from gemcitabine-induced apoptosis.

**Figure 6:**
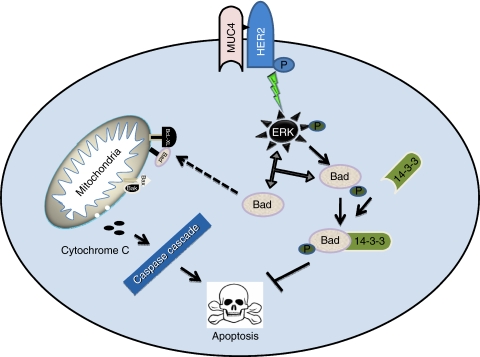
Proposed model for possible mechanism of MUC4-mediated resistance to apoptosis. In a viable cell, the pro-apoptotic Bcl-2 family members (Bax, Bak) and BH3-only proteins, such as Bad, are antagonized by anti-apoptotic members, such as Bcl-X_L_, Bcl-2. In response to an apoptotic stimulus, Bad are activated and prevent anti-apoptotic Bcl-2 members from inhibiting pro-apoptotic members. Pro-apoptotic members then form pores into the mitochondrial membrane and release pro-apoptotic factors, such as cytochrome *c* into the cytosol, which subsequently activates the caspase cascade leading to apoptosis. In response to the gemcitabine treatment in CD18/HPAF/Scr MUC4-expressing pancreatic cancer cells, MUC4 phosphorylates anti-apoptotic protein Bad through MUC4-HER2-ERK-mediated pathway. Phosphorylation of Bad facilitates its binding with scaffolding protein 14-3-3 and, thereby, inhibits translocation of Bad to the mitochondria to deactivate the anti-apoptotic protein Bcl-X_L_. These findings suggest that MUC4-mediated increased phosphorylation of Bad through HER2/ERK pathway might be responsible to protect pancreatic cancer cells from the gemcitabine-induced apoptosis.

Our recent studies have revealed that MUC4 interacts with HER2, a member of EGF receptor family and regulates its expression by post-translational mechanisms (Chaturvedi *et al*, 2008). In addition, a recent study has shown that anti-apoptotic effect of rat Muc 4 is independent of ErbB2/HER2 in A375 melanoma and MCF-7 breast cancer cells, whereas dependent on ErbB2 in JIMT-1 breast cancer cells (Workman *et al*, 2009). Our data showed increased expression and activation of HER2 in CD18/HPAF/Scr pancreatic cancer cells in response to gemcitabine treatment. Earlier studies have shown that MUC4-HER2 interaction subsequently leads to activation of ERK (Chaturvedi *et al*, 2008). We have also shown increased activation of ERK in response to gemcitabine treatment of CD18/HPAF/Scr cells. Further, ERK activation has been shown to protect prostate cancer cells from apoptosis by phosphorylating apoptotic protein Bad (Sastry *et al*, 2006). These observations suggest that activation of anti-apoptotic pathway through MUC4-HER2-ERK-mediated pathway might be responsible for contribution of resistance to chemotherapeutic agent gemcitabine. A schematic model (Figure 6) has been proposed to depict the possible mechanism of MUC4-mediated inhibition of apoptosis in pancreatic cancer cells in response to gemcitabine treatment.

In conclusion, our data provide the first evidence that MUC4 imparts resistance to gemcitabine in pancreatic cancer cells. We showed that MUC4 protects CD18/HPAF pancreatic cancer cells from gemcitabine-induced apoptosis. Furthermore, inhibition of apoptosis was associated with increased phosphorylation of HER2 and ERK. Activated ERK then deactivates pro-apoptotic protein Bad by phosphorylation and, thereby, protects cells from apoptosis. These findings indicate that the overexpression of MUC4 confers resistance to anti-cancer agent gemcitabine. In the future, it will be of interest to examine the effect of gemcitabine treatment on MUC4-expressing and non-expressing pancreatic cancer cell lines *in vivo* to support the pathogenic relevance of MUC4 with the acquisition of resistance to chemotherapeutics.
